# Two new species of *Sinosmylites* Hong (Neuroptera, Berothidae) from the Middle Jurassic of China, with notes on Mesoberothidae

**DOI:** 10.3897/zookeys.130.1418

**Published:** 2011-09-24

**Authors:** Vladimir N. Makarkin, Qiang Yang, Dong Ren

**Affiliations:** 1 College of Life Sciences, Capital Normal University, Beijing, 100048, China; 2 Institute of Biology and Soil Sciences, Far Eastern Branch of the Russian Academy of Sciences, Vladivostok, 690022, Russia

**Keywords:** Neuroptera, Berothidae, Mesoberothidae, Daohugou, China, Middle Jurassic

## Abstract

Two new species of the genus *Sinosmylites* Hong are described from the Middle Jurassic locality at Daohugou (Inner Mongolia, China): *Sinosmylites fumosus* **sp. n.** and *Sinosmylites rasnitsyni* **sp. n.** This is the oldest known occurrence of the family Berothidae. The berothid affinity of this genus is confirmed by examination of the hind wing venation characteristic of the family. The Late Triassic family Mesoberothidae may represent an early group of Berothidae.

## Introduction

Today, the Berothidae (including Rhachiberothinae) is a small neuropteran family comprised of about 100 species discontinuously distributed mainly across tropical and warm-temperate regions of the world (Aspöck 1986; Aspöck and Aspöck 1997; Aspöck and Nemeschkal 1998). Their larvae are thought to be associated with termite nests, where they feed on termites (e.g., Tauber et al. 2003); however, this habit is only documented in the North American genus *Lomamyia* Banks (Johnson and Hagen 1981; Faulkner 1992).

Currently, 34 fossil berothid species have been described from various localities (listed in Table 1; others are described but unnamed, only illustrated or represented by larvae). The family was previously only known tentatively from the Jurassic: Grimaldi and Engel (2005) and Engel and Grimaldi (2008) considered the Jurassic / Early Cretaceous Mesithonidae as possible primitive Berothidae or ‘stem-group berothids’. Formerly, berothids were believed to be a very ancient family, even when it almost entirely lacked a known fossil record. Tillyard (1932) stated, characteristically: “on the totality of characters, it would now appear as if the Berothidae are the oldest existing family of Planipennia [=Neuroptera].” (p. 29). Here, we describe two new species of the genus *Sinosmylites* Hong, 1983 from the Middle Jurassic of China, which we confidently assign to the family Berothidae based on characters of the hind wing. We compare the forewing venation of this genus and those of the Late Triassic Mesoberothidae, and by their similarities confirm the great antiquity of the berothid lineage.

**Table 1.** A list of known fossil Berothidae.

	Species	Age	Locality	References
1	Berothidae gen. et sp. n.	Early Cretaceous (Early Berriasian)	Durlston Bay, England (Lulworth Formation)	Jepson et al. submitted
2	*Banoberotha enigmatica* Whalley, 1980	Early Cretaceous (Valanginian/Hauterivian)	Lebanese amber (Jezzine)	Whalley 1980
3	*Paraberotha acra* Whalley, 1980	Early Cretaceous (Valanginian/Hauterivian)	Lebanese amber (Jezzine)	Whalley 1980; Nel et al. 2005
4	Berothidae indet. (larva)	Early Cretaceous (Valanginian/Hauterivian)	Lebanese amber (Jezzine)	Whalley 1980: figs 9, 10
5	*Chimerhachiberotha acrasarii* Nel et al., 2005	Early Cretaceous (Valanginian/Hauterivian)	Lebanese amber (Jezzine)	Nel et al. 2005
6	*Raptorapax terribilissima* Petrulevicius et al., 2010	Early Cretaceous (Neocomian)	Lebanese amber (Houarij)	Petrulevicius et al. 2010
7	*Spinoberotha mickaelacrai* Nel et al., 2005	Early Cretaceous (Barremian/Aptian)	Lebanese amber (Hammana)	Nel et al. 2005
8	*Oloberotha sinica* Ren et Guo, 1996	Early Cretaceous (Barremian)	Yixian Formation, China	Ren and Guo 1996
9	*Araripeberotha fairchildi* Martins-Neto et Vulcano, 1990	Early Cretaceous (Late Aptian)	Crato Formation, Brazil	Martins-Neto and Vulcano 1990
10	*Caririberotha martinsi*Martins-Neto & Vulcano, 1990	Early Cretaceous (Late Aptian)	Crato Formation, Brazil	Martins-Neto and Vulcano 1990
11	Berothidae indet.	Early Cretaceous (Early Aptian)	Spanish amber (El Sophao)	Peñalver and Delclòs 2010
12	*Alboberotha petrulevicii* Nel et al., 2005	Early Cretaceous (Late Albian)	Archingeay, France	Nel et al. 2005
13	*Eorhachiberotha burmitica* Engel, 2004	Early Cretaceous (Late Albian)	Burmese amber	Engel 2004
14	*Dasyberotha eucharis* Engel et Grimaldi, 2008	Early Cretaceous (Late Albian)	Burmese amber	Engel and Grimaldi 2008
15	*Ethiroberotha elongata* Engel et Grimaldi, 2008	Early Cretaceous (Late Albian)	Burmese amber	Engel and Grimaldi 2008
16	*Haploberotha persephone* Engel et Grimaldi, 2008	Early Cretaceous (Late Albian)	Burmese amber	Engel and Grimaldi 2008
17	*Iceloberotha kachinensis* Engel et Grimaldi, 2008	Early Cretaceous (Late Albian)	Burmese amber	Engel and Grimaldi 2008
18	*Iceloberotha simulatrix* Engel et Grimaldi, 2008	Early Cretaceous (Late Albian)	Burmese amber	Engel and Grimaldi 2008
19	*Jersiberotha myanmarensis* Engel et Grimaldi, 2008	Early Cretaceous (Late Albian)	Burmese amber	Engel and Grimaldi 2008
20	*Jersiberotha tauberorum* Engel et Grimaldi, 2008	Early Cretaceous (Late Albian)	Burmese amber	Engel and Grimaldi 2008
21	*Scoloberotha necatrix* Engel et Grimaldi, 2008	Early Cretaceous (Late Albian)	Burmese amber	Engel and Grimaldi 2008
22	*Systenoberotha magillae* Engel et Grimaldi, 2008	Early Cretaceous (Late Albian)	Burmese amber	Engel and Grimaldi 2008
23	*Telistoberotha libitina* Engel et Grimaldi, 2008	Early Cretaceous (Late Albian)	Burmese amber	Engel and Grimaldi 2008
24	Berothidae indet. (larva)	Early Cretaceous (Late Albian)	Burmese amber	Engel and Grimaldi 2008: figs 42, 43.
25	*Retinoberotha stuermeri* Schlüter, 1978	Late Cretaceous (Cenomanian)	Bezonnais, France	Schlüter 1978
26	*Plesiorobius sibiricus* Makarkin, 1994	Late Cretaceous (Cenomanian)	Obeshchayushchiy, NE Siberia (Ola Formation)	Makarkin 1994
27	*Jersiberotha luzzii* Grimaldi, 2000	Late Cretaceous (Turonian)	Raritan (New Jersey) amber	Grimaldi 2000
28	*Jersiberotha similis* Grimaldi, 2000	Late Cretaceous (Turonian)	Raritan (New Jersey) amber	Grimaldi 2000
29	*Nascimberotha picta* Grimaldi, 2000	Late Cretaceous (Turonian)	Raritan (New Jersey) amber	Grimaldi 2000
30	*Rhachibermissa phenax* Engel et Grimaldi, 2008	Late Cretaceous (Turonian)	Raritan (New Jersey) amber	Grimaldi 2000
31	*Rhachibermissa splendida* Grimaldi, 2000	Late Cretaceous (Turonian)	Raritan (New Jersey) amber	Grimaldi 2000
32	*Plesiorobius* cf. *canadensis*	Late Cretaceous (Santonian)	Yantardakh, N Siberia	Makarkin 1994
33	*Plesiorobius canadensis* Klimaszewski et Kevan, 1986	Late Cretaceous (Campanian)	Canadian amber	Klimaszewski and Kevan 1986
34	*Albertoberotha leuckorum* McKellar et Engel, 2009	Late Cretaceous (Campanian)	Canadian amber	McKellar and Engel 2009
35	Berothidae indet.	Late Cretaceous (Campanian)	Canadian amber	Engel and Grimaldi 2008: fig. 41
36	*Oisea celinea* (Nel et al., 2005)	Early Eocene	Oise amber, France	Nel et al. 2005
37	*Microberotha macculloughi* Archibald et Makarkin, 2004	Early Eocene	Hat Creek amber, British Columbia	Archibald and Makarkin 2004
38	*Whalfera venatrix* (Whalley, 1983)	Late Eocene	English amber	Whalley 1983
39	*Proberotha prisca* Krüger, 1923	Late Eocene	Baltic amber	Krüger 1923
40	*Whalfera wiszniewskii* Makarkin et Kupryjanowicz, 2010	Late Eocene	Baltic amber	Makarkin and Kupryjanowicz 2010
41	Berothidae indet.	Late Eocene	Baltic amber	Bachofen-Echt 1949: fig. 122
42	Berothidae indet.	Late Eocene	Baltic amber	Weitschat and Wichard 1998: pl. 55, figs a, b
43	Berothinae indet.	Late Eocene	Baltic amber	MacLeod and Adams 1967
44	Berothinae indet. (larva)	Late Eocene	Baltic amber	Janzen 2002: fig. 58
45	Berothinae indet. (larva)	Late Eocene	Baltic amber	V.Makarkin, S.Wedmann, T.Weiterschan (ongoing research)
46	Berothinae indet. (larva)	Late Eocene	Rovno amber, Ukraine	E.Perkovsky, V.Makarkin (ongoing research)

## Material and methods

This study is based on three specimens collected from Daohugou Village (Shantou Township, Ningcheng County, Inner Mongolia, China) and housed in the Key Laboratory of Insect Evolution and Environmental Changes, College of Life Sciences, Capital Normal University, Beijing, China (CNUB; Dong Ren, curator). These insect-bearing beds are here considered as belonging to the Jiulongshan Formation and are dated Bathonian, Middle Jurassic (Gao and Ren 2006).

Specimens were examined using a Leica MZ12.5 dissecting microscope; line drawings were prepared with CorelDraw 12 graphics software with the aid of Adobe Photoshop; photographed by a Nikon SMZ1000 stereomicroscope.

Venational terminology principally follows Comstock (1918) as modified by Oswald (1993) and Archibald and Makarkin (2006). Berothid wings possess cross-venation basically similar to that of Hemerobiidae, arranged in four (incomplete) gradate series in both families. Consequently, we adopt Oswald’s (1993) designation of crossveins: principal crossveins are designated by the longitudinal veins which they connect and numbered by the gradate series to which they belong in sequence from the wing base, e.g., 1a1-a2, the crossvein connecting 1A and 2A in the first gradate series; 2m-cu, the crossvein connecting M and Cu in the second gradate series; 2icu, the intracubital crossvein (i.e., between CuA and CuP) in the second gradate series; and 4rs1-rs2, the crossvein between Rs1 and Rs2 in the fourth gradate series. Terminology of wing spaces mainly follows Oswald (1993).

Abbreviations used in the text and figures are as the follows: 1A–3A, first to third anal veins; CuA, CuP, anterior and posterior branches of the cubital vein (Cu); MA, MP, anterior and posterior branches of the medial vein (M); R1, anterior branch of the radial vein (R); Rs1, most proximal branch of the radial sector (Rs); Rs2, branch of the radial sector located distal to Rs1; Rs3, branch of the radial sector located distal to Rs2; Sc, subcostal vein.

## Taxonomy

### Family Berothidae Handlirsch, 1906

#### Genus Sinosmylites

Hong, 1983 sit. n.

http://species-id.net/wiki/Sinosmylites

Sinosmylites Hong, 1983: 94, 198 [Osmylitidae]; Ren et al. 1995: 101 [?Osmylidae]; Ren and Guo 1996: 466 [‘osmylid-like’ Neuroptera]; Makarkin and Archibald 2005: 15, 16, 18, 19 [probably Prohemerobiidae]; Yang et al. 2010: 177 [Osmylidae].

##### Type species.

*Sinosmylites pectinatus* Hong, 1983, by original designation.

##### Diagnosis.

Forewing: costal space strongly narrowed basally; humeral veinlet not recurrent and branched; Sc, R1 fused distally; Sc+R1 with 9-11 veinlets, mostly simple; all subcostal veinlets simple; M forked far distal to origin of Rs; CuA pectinate, with seven branches; few crossveins in radial space arranged mainly in 1-2 ‘inner’ gradate series.

##### Species included.

Three species from the Middle Jurassic of China (Jiulongshan Formation): *Sinosmylites pectinatus* (Liaoning Province), *Sinosmylites fumosus* sp. n. and *Sinosmylites rasnitsyni* sp. n. (Inner Mongolia).

##### Comments.

The venation of these two new species is very similar to that of *Sinosmylites pectinatus*. The latter species is represented by a nearly complete forewing (Hong 1983). Unfortunately, however, it is quite poorly figured, and its type is now lost. Nevertheless, all main features of *Sinosmylites fumosus* sp. n. and *Sinosmylites rasnitsyni* sp. n. forewings agree well with those confirmed of *Sinosmylites pectinatus*, i.e., similar size (length 5.5 mm in *Sinosmylites pectinatus*; about 6.5 mm in *Sinosmylites fumosus* sp. n.; 6.7 mm in *Sinosmylites rasnitsyni* sp. n.), coloration (a single, more or less fuscous color), and the venational character states are as provided in the generic diagnosis. The few differences between the type species and the two new species (e.g., the presence of two ‘inner’ gradate series, and the CuP twice forked in *Sinosmylites pectinatus*) are at most specific.

#### Sinosmylites rasnitsyni sp. n.

urn:lsid:zoobank.org:act:8851F470-C9D3-4E72-87AA-B8D8639DB16F

http://species-id.net/wiki/Sinosmylites_rasnitsyni

Figs 1 –3

##### Diagnosis.

Differs from both other species of *Sinosmylites* by more closely spaced subcostal veinlets, and more deeply forked CuP.

##### Description.

Body indistinctly preserved. Antennae moniliform, incomplete; preserved segments transverse (wider than long). Prothorax short. Mesonotum of usual neuropteran morphology. Legs covered with short hairs; fore-, mid-legs relatively short; hind-leg tibia long; fore-, hind-leg basitarsus longest segment of tarsus. Abdomen very poorly preserved.

Forewing with broad-rounded apex, 6.7 mm long, 3.0 mm wide. Costal space moderately broad, strongly dilated at proximal 1/5 of wing length, narrowed basally. Subcostal veinlets simple, regularly arranged, closely spaced. Sc distally fused with R1 far from wing apex; Sc+R1 with 9-11 simple veinlets. Subcostal space broad, with one basal crossveins located immediately after origin of Rs. R1 space narrower than subcostal space; three widely-spaced crossveins before fusion of Sc, R1, one after. Rs with 11 (right wing), 10 (left wing) parallel pectinate regularly-spaced branches; six proximal branches with 2-4 terminal forks, other branches once forked. Rs1 originating near origin of Rs. M appears fused basally for short distance; forked much distal to origin of Rs1. MA, MP almost parallel, distally with one, two quite long forked branches respectively. Cu divided into CuA, CuP proximal to origin of Rs. CuA pectinate, with 7 branches, some once forked. CuP once deeply forked. Anal veins very poorly preserved; 1A, 2A apparently once deeply forked each; 3A simple. Four gradate series of crossveins posterior to stem of Rs partly preserved (series 1-4 of Oswald 1993). First series: crossvein 1r-m (located at origin of Rs). Second series: crossveins 2icu (connecting CuA, anterior branch of CuP), 2icup (between branches of CuP). Third (‘inner’) series: six crossveins preserved (between Rs1, Rs8). Fourth (‘outer’) series: four irregularly-spaced crossveins preserved (between Rs2, CuA). Wing one color, slightly fuscous. Veins dark brown as preserved.

Hind wing poorly preserved, approximately 6.5 mm long, 2.6 mm wide. Costal space narrow, distally only slightly dilated. Subcostal veinlets simple, rather closely spaced. Sc distally fused with R1 far from wing apex; Sc+R1 with 13 simple veinlets. Subcostal space relatively narrow; no crossveins detected. R1 space broad, dilated basally; two crossveins before fusion of Sc, R1, one after. Rs originating far from wing base, with eight branches, each forked distally 1-3 times except Rs1 which deeply forked four times. Fork of M not detected. MA once forked distally; MP dichotomously branched distally. CuA long, almost parallel to hind margin, its branches poorly preserved. CuA space relatively broad. CuP fragmentary preserved, quite short. Anal veins not preserved. Crossveins posterior to stem of Rs not detected except one distal between MP, CuA (4m-cu).

**Figure 1.**
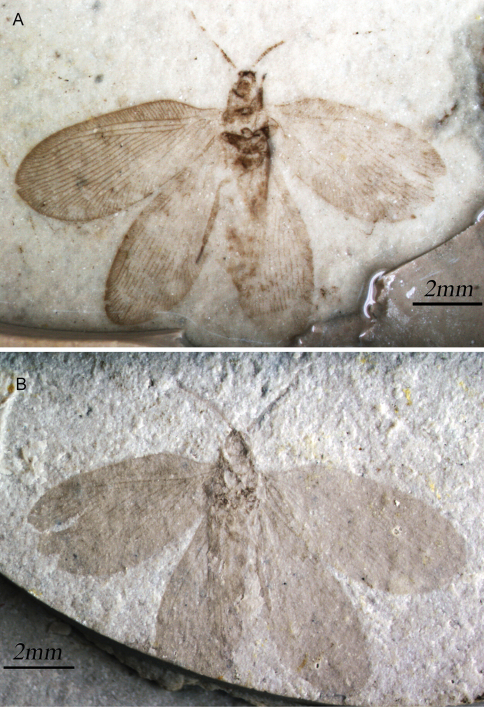
*Sinosmylites rasnitsyni* sp. n. Photograph of the holotype **A** part (CNU-NEU-NN2011002P; in alcohol) **B** counterpart (CNU-NEU-NN2011002C; dry).

**Figure 2.**
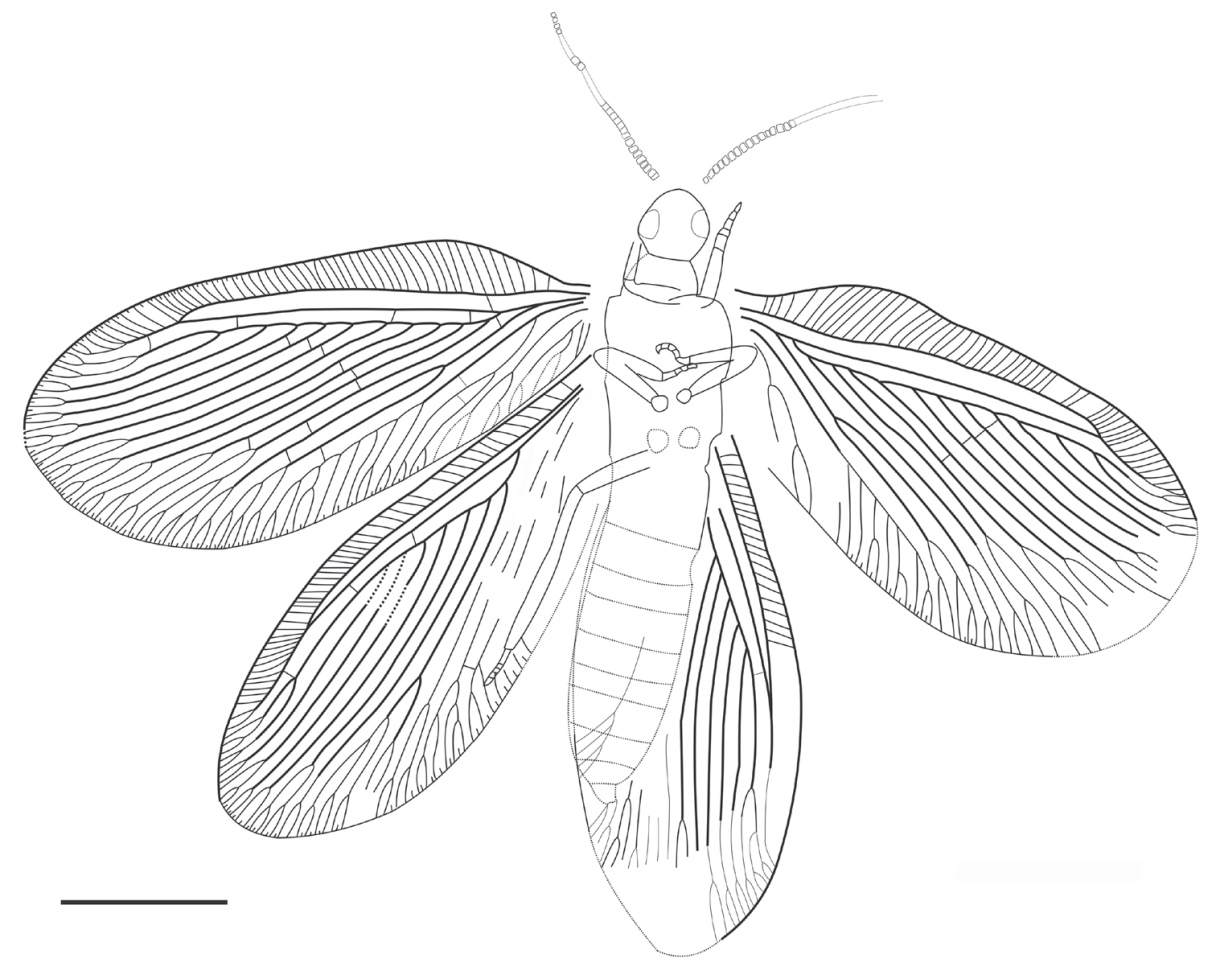
*Sinosmylites rasnitsyni* sp. n. Drawing of the holotype CNU-NEU-NN2011002P. Scale bar is 2 mm.

**Figure 3.**
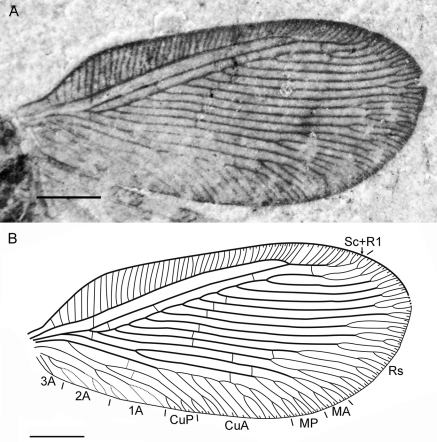
*Sinosmylites rasnitsyni* sp. n. Forewing of the holotype CNU-NEU-NN2011002P (converted to the right). **A** photograph **B** drawing. Scale bar is 1 mm.

##### Material.

Holotype CNU-NEU-NN2011002P (part), CNU-NEU-NN2011002C (counterpart), deposited in CNUB. A nearly complete specimen.

##### Type locality and horizon.

Daohugou Village, Shantou township, Ningcheng county, Inner Mongolia, China. Jiulongshan Formation, Middle Jurassic.

##### Etymology.

The species is named in honor of the distinguished Russian paleoentomologist Prof. Alexandr Pavlovich Rasnitsyn.

#### Sinosmylites fumosus sp. n.

urn:lsid:zoobank.org:act:544F55FC-01D7-4655-BD4F-CBEE79EA247F

http://species-id.net/wiki/Sinosmylites_fumosus

Figs 4A, B

##### Diagnosis.

Differs from *Sinosmylites pectinatus* by CuP once forked (twice forked in *Sinosmylites pectinatus*), by presence of one ‘inner’ gradate series of crossveins (two in *Sinosmylites pectinatus*) (see differences from *Sinosmylites rasnitsyni* sp. n. under that species.).

##### Description.

Forewing with broad-rounded apex, about 6.0 mm long (as preserved, estimated complete length about 6.5 mm), 2.6 mm wide. Costal space moderately broad, most dilated at proximal 1/5 of wing length. Subcostal veinlets simple, regularly arranged, less closely spaced than in previous species. Sc distally fused with R1 far from wing apex; Sc+R1 with nine veinlets (eight simple, one forked). Subcostal space broad, with two basal crossveins. R1 space nearly as wide as subcostal space; six crossveins before fusion of Sc and R1, one after. Rs with nine pectinate, regularly spaced branches; four proximal-most branches with 2-4 terminal forks, other branches once forked. Rs1 originating at some distance from origin of Rs. M not fused basally; forked much distal to origin of Rs1. MA, MP almost parallel, distally with one (simple) , two (one simple) branches respectively. Cu divided into CuA, CuP proximal to origin of Rs. CuA pectinate, with 7 branches; proximal-most branch once forked. CuP once deeply forked. Anal veins incompletely preserved; 1A with single marginal fork; 2A with two marginal short branches; 3A very incomplete, with single fork preserved. Four gradate series of crossveins posterior to stem of Rs, all incomplete. First series consists of three crossveins: 1r-m (located at origin of Rs), 1m-cu, 1a1-a2 (longer than previous); Second series includes two crossveins: 2m-cu (connecting MP, CuA), 2icu (connecting CuA, anterior branch of CuP). Third (‘inner’) series with six crossveins (3rs-rs7, 3rs5-rs4 to 3rs2-rs1; two between Rs3, Rs2). Fourth (‘outer’) series with five crossveins (from 4rs2-rs1 to 4m-cu; two between Rs1, MA). Wing one color, fuscous. Veins mainly dark brown as preserved.

**Figure 4.**
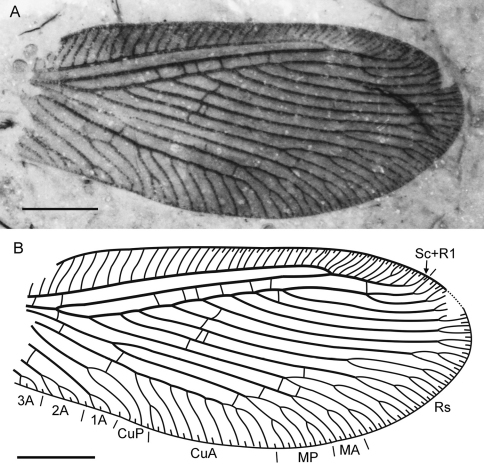
*Sinosmylites fumosus* sp. n. Holotype CNU-NEU-NN2011003, the forewing. **A** photograph **B** drawing. Scale bar is 1 mm.

##### Type material.

Holotype CNU-NEU-NN2011003, deposited in CNUB. A nearly complete forewing.

##### Type locality and horizon.

Daohugou Village, Shantou township, Ningcheng county, Inner Mongolia, China. Jiulongshan Formation, Middle Jurassic.

##### Etymology.

From the Latin *fumosus*, smoked, in reference to the coloration of wings.

#### Sinosmylites sp.

http://species-id.net/wiki/Sinosmylites

Figs 5A, B

##### Description.

Hind wing approximately 6.5 mm long, 2.7 mm wide. Humeral lobe not extended; frenulum poorly-developed consisting of few bristles. Costal space narrow, dilated towards apex. Subcostal veinlets simple, more closely-spaced apically. Sc distally fused with R1 far from wing apex; Sc+R1 with seven long veinlets (one forked). Subcostal space relatively broad, with one basal crossveins. R1 space nearly as wide as subcostal space; four crossveins before fusion of Sc, R1. Rs with seven pectinate, regularly spaced branches; one branch deeply forked. Rs1 originating at some distance from origin of Rs. Proximal crossvein m-r long, connecting Rs1 near its origin with M. M forked distal to origin of Rs1. MA, MP almost parallel, distally with few branches. CuA long, slightly incurved, in general parallel to hind margin, with nine forkes branches originated at angle >45 degrees, one simple branch. CuP short, with two branched preserved. 1A–3A not preserved. Crossvein between CuA, 1A (or CuP). One crossvein between Rs, Rs6 in ‘inner’ gradate series (possibly anomalous). Six crossveins (from Rs4 to CuA) in ‘outer’ gradate series preserved. Wing one color, fuscous. Veins appear mainly dark brown.

**Figure 5.**
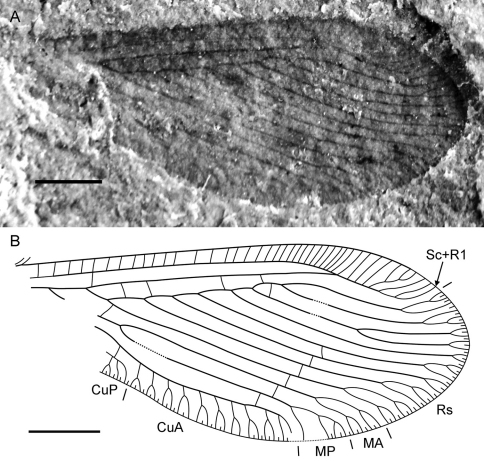
*Sinosmylites* sp. Specimen CNU-NEU-NN2011004, the hind wing. **A** photograph **B** drawing. Scale bar is 1 mm.

##### Material examined.

Specimen CNU-NEU-NN2011004, deposited in CNUB. A nearly complete hind wing.

##### Type locality and horizon.

Daohugou Village, Shantou township, Ningcheng county, Inner Mongolia, China. Jiulongshan Formation, Middle Jurassic.

##### Comments.

The venation of this hind wing is typical for Berothidae. In particular, the configuration of CuA is characteristic of this family; although this also occurs in the Nevrorthidae, nevrorthid venation is otherwise dissimilar. Also, the basal crossvein between R and M systems is straight, perpendicular to connecting veins; this is characteristic of all Berothidae except Rhachiberothinae. In the vast majority of extant Berothidae, the complete CuP is lost, but the basal or/and distal parts there are often present. CuP is entirely lost in some genera, both fossil (e.g., *Microberotha macculloughi* Archibald and Makarkin, 2004) and extant (e.g., *Cyrenoberotha* MacLeod and Adams, 1968, *Berlekrumyia* Aspöck and Aspöck, 1988). Therefore, it is hard to determine which vein is preserved in this hind wing, the distal part of CuP or 1A (see Fig. 5B, labeled *CuP*), as the proximal portion of the wing is not preserved. We tentatively consider this vein to be CuP.

This specimen is tentatively assigned to *Sinosmylites*. The hind wings of *Sinosmylites rasnitsyni* sp. n., the only species of the genus in which these are known,are quite poorly preserved and their venation does not enable its generic character states to be determined with confidence. However, this hind wing shares similar size, coloration, and venation (e.g., Sc and R1 are fused; Sc+R1 with many branches; several crossveins between R1 and Rs; the same configuration of the Rs branches) with the forewings of *Sinosmylites* species. Therefore, this generic affinity is most likely.

## Discussion

As the type species of the genus *Sinosmylites* is represented by a single forewing, its family affinity cannot be confidently determined. Its venation is more or less similar to that of such families as Berothidae, Sisyridae, Mesoberothidae, Archeosmylidae, and Prohemerobiidae, all except the first two are extinct and poorly understood. Their forewing venations show superficial similarities in the structure of the costal space (narrowed basally), Sc fused with R1 (only convergent distally but not fused in Prohemerobiidae), similar (in general) branching of Rs, M, and Cu, and their sparse cross-venation. One feature occurs rarely (if at all) in these taxa, i.e., the strongly pectinate CuA, complicating family determination. Therefore, based solely on the forewing, it may be theoretically associated with most of these families, at least provisionally. Fortunately, one of two new species described herein bears its hind wing, although poorly preserved. Its structure indicates that the berothid affinity of *Sinosmylites* is most probable, as its general venation does not conflict with that of Berothidae, and the presence of the long CuA running nearly parallel to the hind margin characteristic of the family. Moreover, the berothid affinity of a better preserved hind wing (“*Sinosmylites* sp.”) is doubtless, as all of its character states are characteristic only of the Berothidae.

The forewing venation of *Sinosmylites* differs rather greatly from that of the vast majority of extant (advanced) genera of Berothidae. Particularly, M is forked distinctly more distally than in most berothid genera (including Cretaceous genera: see Engel and Grimaldi 2008), and CuP is strongly pectinate as rarely occurs in Berothidae. It appears most closely related to an Early Cretaceous genus from the Purbeck Limestone Group, which is represented by two nearly complete forewings (Jepson et al. submitted). These two genera share common venational character states (including a pectinate CuA and distal forking of M), but the Purbeck genus is much smaller (forewing length 3.7-3.8 mm) and in general has simpler venation. *Sinosmylites* is quite similar also to *Banoberotha enigmatica* Whalley, 1980 from the Early Cretaceous of Lebanese amber by the very similar outline of the costal space, simple veinlets, and M forked much distally to the origin of Rs, but otherwise their venation is different.

The hind wing venation of “*Sinosmylites* sp.” appears amazingly modern. Even if our generic attribution turns out to be incorrect, its berothid family affinity is doubtless.

Triassic berothid-like taxa have been treated as belonging to the family Mesoberothidae. This taxon was created by Riek (1955) as the family Proberothidae for two genera from the Late Triassic Mount Crosby Formation in Australia, *Proberotha* Riek, 1955 and *Proberothella* Riek, 1955. The name Proberothidae was later replaced with Mesoberothidae by Carpenter (1991) as its type genus turned out to be a junior homonym of *Proberotha* Krüger, 1923. The family has never been revised, with Riek’s two genera its only members (Jell 2004). It has remained, as Carpenter (1992: p. 349) noted, a “little-known family”.

*Mesoberotha* is represented by a single forewing specimen of *Mesoberotha superba* Riek, 1955, whose venation is similar to that of *Sinosmylites* (especially *Sinosmylites fumatus* sp. n., known from a better preserved forewing), i.e., the costal space is similarly constructed, narrowed basally; Sc and R1 are fused; the subcostal space is relatively broad; few crossveins are present, most of which are arranged in a gradate series of crossveins; M, Cu, CuA and CuP are branched in a similar manner; 1A with only marginal (shallow) branches. As mentioned above, this genus cannot be assigned to a particular family with confidence based only on characters of the forewing. However, the similarity of the venation between *Sinosmylites* and *Mesoberotha* is so distinct as to strongly suggest that *Mesoberotha* may belong to Berothidae, and that Mesoberothidae is, therefore, a synonym of Berothidae. However, it is necessary to find and examine the *Mesoberotha* hind wing in order to test this hypothesis.

Riek (1955) believed that the family Mesoberothidae “seems to be directly ancestral to the Berothidae.” (p. 674). Similarly, Mesoberothidae was referred by Engel and Grimaldi (2008) to the epifamily Mantispoidae (or ‘dilarid clade’) of the superfamily Hemerobioidea, which also contains Berothidae, Rhachiberothidae, Mantispidae, and Dilaridae. Haring and Aspöck (2004) stated that “the Berothidae are the sister group of the ithonid clade + (Mantispidae + (Chrysopidae + Hemerobiidae))” (p. 427). The oldest known taxon of the latter group is the mantispid genus *Liassochrysa* Ansorge and Schlüter, 1990 from the Early Toarcian of Dobbertin (Germany) (Wedmann and Makarkin 2007), and, therefore, the Berothidae must be at least of the same Early Jurassic age. Estimates of divergences times based on molecular analysis indicate that the clade consisting of Berothidae and Mantispidae arose during the Early Triassic (Winterton et al. 2010). Consequently, the Late Triassic Mesoberothidae may well represent an early group of Berothidae.

*‘Archeosmylus’ ?costalis* Riek, 1955 from the same Australian locality as *Mesoberotha* probably also belongs to the Mesoberothidae. Its venation differs not sufficiently from that of *Mesoberotha superba*, and it may possibly belong to this genus. The family affinity of *‘Archeosmylus’ stigmatus* Riek, 1955 is not yet clear.

## Supplementary Material

XML Treatment for Sinosmylites

XML Treatment for Sinosmylites rasnitsyni

XML Treatment for Sinosmylites fumosus

XML Treatment for Sinosmylites
